# Molecular screening indicates high prevalence and mixed infections of Hepatozoon parasites in wild felines from South Africa

**DOI:** 10.4102/jsava.v91i0.2055

**Published:** 2020-11-30

**Authors:** David J. Harris, Dimitra Sergiadou, Ali Halajian, Lourens Swanepoel, Francois Roux

**Affiliations:** 1 CIBIO Research Centre in Biodiversity and Genetic Resources, InBIO, Universidade do Porto, Vila do Conde, Portugal; 2 Department of Biodiversity, University of Limpopo, Sovenga, South Africa; 3 Department of Zoology, University of Venda, Thohoyandou, South Africa; 4 African Institute for Conservation Ecology, Levubu, South Africa; 5 Mpumalanga Tourism and Parks Agency, Lydenburg, Mpumalanga, South Africa

**Keywords:** 18S rRNA, hepatozoonosis, *Panthera*, *Leptailurus*, Caracal, *Felis*, phylogeny

## Abstract

Genetic diversity within partial 18S rRNA sequences from *Hepatozoon* protozoan parasites from wild felines in South Africa was assessed and compared with data from domestic cats to assess patterns of host specificity. Lions, leopards, servals, a caracal and an African wildcat were all positive for parasites of the *Hepatozoon felis*-complex. However, haplotypes were not species-specific, and potential mixed infections were widespread. Additional genetic markers are needed to untangle the extremely complex situation of these parasites in both domestic cats and wild felines in South Africa.

## Introduction

Companion animals can contribute to the spread of pathogens in both humans and wildlife, and identifying pathogens is crucial to understanding disease dynamics. Common companion animals such as dogs and cats play a key role in the epidemiology of various pathogens, acting as both reservoirs and sentinels. Surprisingly therefore, studies of feline vector-borne infections are less prevalent than those of canine vector-borne infections (Pereira et al. 2019). This is despite the fact that cats often spend considerable time outdoors, in areas with high risk of exposure to ticks and other vectors (Persichetti et al. 2018). Of special interest is how host-specific particular pathogens are, or if they can spread to wild felines, which often have high conservation concern.

Amongst pathogens transmitted by ticks, feline hepatozoonosis has been increasingly reported from hosts that include lynx, cheetahs, lions, jaguars, tigers, wildcats and domestic cats (Baneth et al. 1998). Until recently, almost all such parasites were referred as *Hepatozoon felis* (*H. felis*), although a new species was recently described from wildcats, *Hepatozoon silvestris* (Hodžić et al. 2017). Pereira et al. (2019) identified two unrelated genetic lineages within *H. felis*, only one of which was considered to occur in Africa. However, Harris et al. (2019) reported that the situation is even more complex, with diverse lineages in domestic cats from South Africa, and that cats may also be infected by unrelated *Hepatozoon* via trophic interactions. Furthermore, *H. felis*-like lineages have been identified in a genet, *Genetta genetta* (Harris et al. 2017), and a mongoose, *Ichneumia albicauda* (Harris et al. 2018), both from South Africa, highlighting just how convoluted and poorly understood the distribution of these parasites are. In particular, for most wild felines, microscopic studies alone have been used to diagnose hepatozoonosis, and therefore exactly which species of lineages of *Hepatozoon* is found in these hosts is largely unknown. Recently, Viljoen et al. (2020) identified multiple distinct genetic lineages of *H. felis* within caracals from South Africa, whilst Van As et al. (2020), using both molecular and morphological data, described two new species of *Hepatozoon* from African leopards, both of which were embedded within a paraphyletic *H. felis*. Both studies highlight the need for further assessments within the complex.

In this study, we employed a molecular approach to screen 22 samples of wild felids from South Africa for *Hepatozoon* and related parasites. Our molecular approach was similar to a previous approach screening domestic cats for *Hepatozoon* (Harris et al. 2019). In this way, we could determine if the same haplotypes were found in different species within South Africa, to shed light on potential transmission pathways and overall diversity within the *H. felis* species complex.

## Research methods and design

Sampling consisted of specimens collected as road kills, or were from tissue banks from various collections in South Africa. Twenty-two individuals were screened, with muscle and liver tissues being tested (Table 1). The methodology followed the approach used in previous screening studies to extract DNA from apicomplexan parasites using high salt procedures (Harris, Maia & Perera 2012). Briefly, polymerase chain reaction (PCR) amplification of part of the 18S rRNA gene was performed using the primers HepF300 and HepR900 (Ujvari, Madsen & Olssen 2004) with 35 cycles consisting of 95 °C (45 s), 60 °C (45 s) and 72 °C (90 s). Negative and positive controls were run with each reaction, and products sequenced by a commercial company (Genewiz, Germany). Electropherograms were examined by eye and compared with published sequences on GenBank using BLAST. All new sequences were submitted to GenBank

**TABLE 1:** List of positive sample sequences in this study and included in the haplotype network analysis.

Haplo	Sample code	Parasite	Host species	Locality (province)
44	Wca181	*H. felis* complex	*Felis silvestris lybica*	Vivo (Limpopo)
44	Cara118	*H. felis* complex	*Caracal caracal*	Alldays (Limpopo)
27, 47	Leo1116†	*H. felis* complex	*Panthera pardus*	Lydenburg (Mpumalanga)
27, 47	LioM717	*H. felis* complex	*Panthera leo*	Kaapmuiden (Mpumalanga)
28, 47	LeoK1217	*H. felis* complex	*Panthera pardus*	Mpumalanga Province
29, 47	LioM7172	*H. felis* complex	*Panthera leo*	Mpumalanga Province
44, 47	SerS818	*H. felis* complex	*Leptailurus serval*	Secunda (Mpumalanga)
47, 50	SerM17	*H. felis* complex	*Leptailurus serval*	Lydenburg (Mpumalanga)
47, 51	SerM1116	*H. felis* complex	*Leptailurus serval*	Lydenburg (Mpumalanga)
51, 56	LioF817	*H. felis* complex	*Panthera leo*	Mpumalanga Province
51, 56	SerS175	*H. felis* complex	*Leptailurus serval*	Secunda (Mpumalanga)

Note: A full list of samples including those from GenBank is given in Online Appendix 1.

**FIGURE 1:**
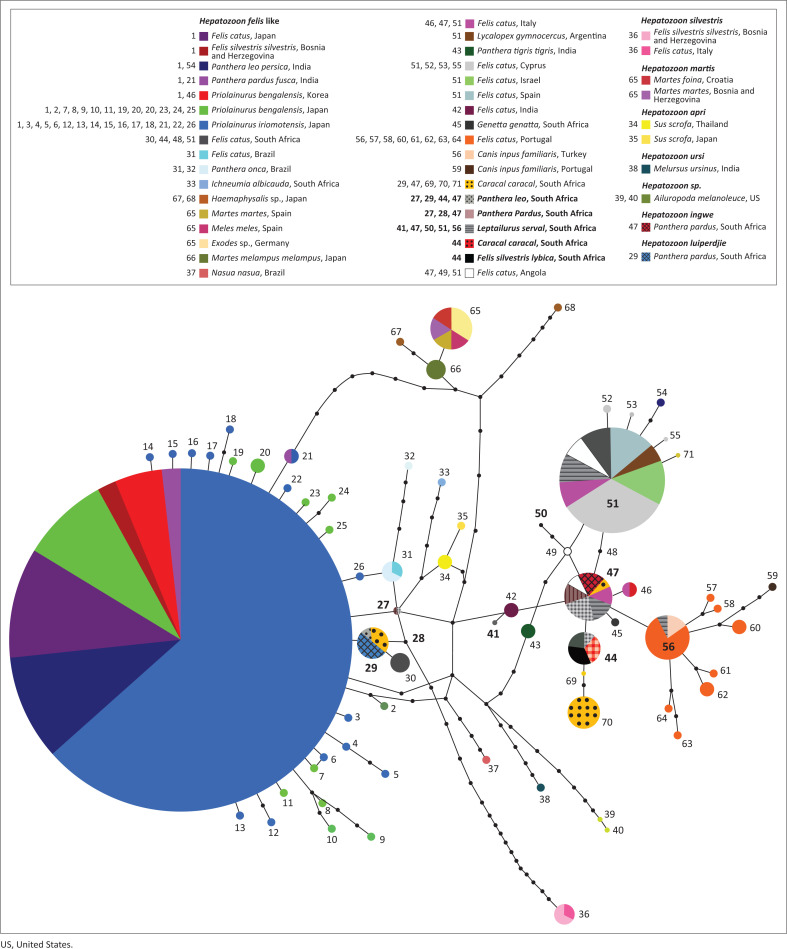
Network showing relationships between haplotypes of *Hepatozoon felis* and other closely related species. Size of circles is proportional to the numbers of haplotypes. The new samples analysed in this study are shown in bold.

Haplo, haplotype code corresponding to Figure 1.

† , Indicates road-killed specimen.

New sequences were aligned against published data using the ClustalW software implemented in Geneious 4.8.5 (Biomatters Ltd). Haplotype phase was estimated using the PHASE algorithm (Stephens, Smith & Donnelly 2001). Sequences included are given in Online Appendix 1. We estimated a phylogenetic network using the approach implemented in TCS (Posada & Crandall 2000:1657–1659). Although longer fragments were generated in this study, as many of the fragments available on GenBank were much shorter, a final alignment of 255 base pairs was used.

### Ethical considerations

The authors confirm that ethical clearance was not required for this study.

## Results

Of the 22 samples examined, positive amplifications for species of *Hepatozoon* were obtained from nine specimens, including from lions (*Panthera leo*), leopards (*Panthera pardus*), servals (*Leptailurus serval*), a caracal (*Caracal caracal*) and an African wildcat (*F. silvestris lybica*) (Table 1). One specimen of an African wildcat and another of a caracal both gave positive PCR reactions resulting in the same identified haplotype (44, Figure 1). This haplotype was also identified in a lion in this study, and had previously been found in a domestic cat, also from South Africa (Harris et al. 2019). All other specimens gave heterozygotic sequences. Two leopards were positive with haplotypes (47, 27) and (47, 28). Haplotype 47 has been reported from domestic cats in Italy and Angola (Oliveira et al. 2018), and in this study was found in leopards, lions and servals. Haplotype 47 is identical to haplotypes of *Hepatozoon ingwe* (Van As et al. 2020) from leopards for this fragment of 18S rRNA. Haplotypes 27 and 28 had not been reported before, but differed by only one or two nucleotides from the commonest haplotype 1. Two lions were positive with haplotypes (47, 27) and (47, 29). Thus, one lion had identical haplotypes to a leopard, whilst the other showed a similar pattern, differing by a single nucleotide. The third lion that was positive had haplotypes (44, 47), thereby sharing one haplotype with the caracal and wildcat and the second with the other two lions. Haplotype 29 was also recently identified in caracal (Viljoen et al. 2020), and is identical to *H. luiperdije* (Van As et al. 2020) from leopards for this gene fragment. Four servals were positive for haplotypes (47, 50), (47, 51), (41, 47) and (51, 56). Haplotype 56 was previously known only from domestic cats and dogs, in Portugal and Turkey, respectively (Maia et al. 2014a). Haplotype 51 has been reported from cats in Spain, Israel, Italy, Angola and South Africa, and also pampas fox (*Lycalopex gymnocercas*) in Argentina (Giannitti et al. 2012). Haplotypes 50 and 41 are newly identified here for the first time and are so far only known from servals.

## Discussion

Several recent screening approaches have shone light on the genetic diversity within *H. felis* in mammals in Africa. Pereira et al. (2019) identified *H. felis* in 15% of cats from Maio Island, in the Cape Verde archipelago, all of which were identical to a haplotype previously identified in cats from Spain. *Hepatozoon felis* from cats in Angola (Oliveira et al. 2018) also shared this and two other closely related haplotypes (47, 49 and 51, Figure 1). Unexpectedly, in a survey of rodents from Nigeria, *H. felis*-like parasites were also detected in *Rattus rattus* and *Rattus norvegicus* (Kamani et al. 2018), further demonstrating the complexity of the situation, although exact haplotypes were not reported, and thus could not be included in this analysis. Harris et al. (2019) identified divergent haplotypes of *H. felis* in domestic cats from South Africa (30, 44, 48, 51, Figure 1), whilst *H. felis*-like haplotypes were also identified in a genet, *Genetta genetta*, and a white-tailed mongoose, *Ichneumia albicauda*, (45 and 33 in Figure 1, Harris et al. 2017:18). Given the paraphyly of *H. felis*, and the complex relationships with closely related species of *Hepatozoon* – including *H. martes* (65), *H. ursi* (38), the recently described *H. ingwe* and *H. luiperdije* (Van As et al. 2020) and a *Hepatozoon* identified in giant pandas *Ailuropoda melanoleuca* (39, 40) – it is clear that *H. felis* as currently understood is a species complex. What remains uncertain is which lineages might occur in diverse wild felids, and how many species should be recognised within the complex.

Our screening approach indicates that the situation in wild felines is extremely complex. Although lions, leopards, servals, caracals and African wildcats were all infected with *H. felis*-like parasites, the haplotypes identified were not species-specific. Rather, as more data are gathered from different *H. felis* and related species of *Hepatozoon*, the relationships of parasites becomes more confused, with some being found in several different species and in diverse geographical locations. One difficulty with this screening methodology is that it is not possible to distinguish unambiguously between mixed infections and heterozygotic sequences from a single parasite species; multiple 18S rRNA haplotypes were recovered within the whole genome of *Hepatozoon canis* (Léveillé et al. 2019), and even cloning approaches cannot distinguish between these possibilities. However, 18S rRNA is generally a slow-evolving gene, so finding haplotypes that are quite divergent within the same host seems improbable relative to the possibility of mixed infection. The recent identification of mixed infections of *H. ingwe* and *H. luiperdije* (Van As et al. 2020) would further support this. Given the diversity of feline species found in South Africa, it is therefore a reasonable hypothesis that mixed infections are common in this area. Mixed infections of parasites can be more detrimental to a host than single infections, and interactions between parasites within a host can be multifaceted (Johnson, De Roode & Fenton 2015), so this possibility needs further investigation. Predation as a pathway of pathogen spillover from domestic cats to wild felids has been documented in various cases (e.g. Kellner et al. 2018), and would fit within the emerging paradigm of trophic pathways leading to infections from *Hepatozoon* species (e.g. Maia et al. 2014). However, very little is known regarding the tick vectors for these parasites, and how these influence the pathogens observed. Viljoen et al. (2020) identified at least six species of ticks from caracals, adding an additional layer of complexity to an already convoluted puzzle. Clearly, additional genetic markers, other than 18S rRNA, are needed to better resolve the tangled relationships with the *H. felis* complex. However, despite the recent data available from *H. canis* (Léveillé et al. 2019), few markers are available that amplify across species (Harris 2020). Until some new markers are developed, relationships are likely to remain uncertain.

To conclude, screening approaches continue to revolutionise our understanding of parasites of the *H. felis* complex, not only in assisting in the description of new, related species, but also in terms of identifying them in new hosts such as rats, and regarding the intricacy of relationships between genetic lineages. Felines appear to be unusual in terms of the possibility of extensive mixed infections, which can confuse assessments based on either microscopy or genetic screening if not accounted for. Whether some of these lineages can be separated by morphological assessments of gamonts remains to be determined, although *H. ingwe* and *H. luiperdije* (Van As et al. 2020) show distinguishing characteristics in blood smears. Further work is also needed to assess the potential impact on the host of mixed infections. If, as seems likely, different genetic lineages correspond to distinct species, then host specificity is low within wild felines.
